# Optimization of the Ultrasonic-Assisted Extraction Technology of Steroidal Saponins from *Polygonatum kingianum* Collett & Hemsl and Evaluating Its Quality Planted in Different Areas

**DOI:** 10.3390/molecules27051463

**Published:** 2022-02-22

**Authors:** Sen He, Xifu Wang, Jiaqiang Chen, Xiaogang Li, Wen Gu, Fan Zhang, Guanhua Cao, Jie Yu

**Affiliations:** 1 School of Chinese Materia Medica and Yunnan Key Laboratory of Southern Medicine Utilization, Yunnan University of Chinese Medicine, Kunming 650500, China; senhe2019@outlook.com (S.H.); a3140208182@163.com (X.W.); sunbelt123@163.com (J.C.); cgh20031695@163.com (X.L.); w1927607391@163.com (W.G.); m2423511271@163.com (F.Z.); 2 Yunnan Key Laboratory for Dai and Yi Medicines, Yunnan University of Chinese Medicine, Kunming 650500, China

**Keywords:** *Polygonatum kingianum*, steroidal saponins, dioscin, diosgenin, ultrasonic-assisted extraction (UAE), response surface methodology (RSM), high-performance liquid chromatography (HPLC)

## Abstract

*Polygonatum kingianum* Collett & Hemsl is one of the famous traditional Chinese herbs with satisfactory therapeutic effects on invigorating Qi, nourishing Yin and moistening lungs, in which steroidal saponins are one class of important active substances. The main purpose is to determine the optimal extraction technology of steroidal saponins and evaluate the quality of *P. kingianum* planted in five different areas. The optimal ultrasonic-assisted extraction (UAE) technology was established by using single-factor experiments and the response surface methodology (RSM), and the determination method of high-performance liquid chromatography (HPLC) for dioscin and diosgenin, two primary types of acid-hydrolyzed steroidal saponins, was constructed with good linear range and precision. The results showed that UAE was an efficient extraction method for steroidal saponins, and the extraction yield was significantly affected by the liquid-solid ratio. The optimal extraction technology was generated following a liquid-solid ratio of 10:1 (mL/g), an ethanol concentration of 85% (*v*/*v*), an extraction time of 75 min, an extraction temperature of 50 °C and three extractions, of which these parameters were in line with the predicted values calculated by RSM. Considering only dioscin and diosgenin, the quality of *P. kingianum* planted at five sample plots presented non-significant difference. However, the content of diosgenin in Pingbian Prefecture (PB) was higher than that of the other four areas with a value of 0.46 mg/g. Taken together, the optimal UAE technology for *P. kingianum* steroidal saponins was determined via RSM. The quality evaluation revealed that there was a non-significant difference among *P. kingianum* planted in different areas based on the contents of the sum of dioscin and diosgenin. This work has important reference value for the exploitation and utilization of *P. kingianum.*

## 1. Introduction

*Polygonatum kingianum* Collett & Hemsl is one of the most important traditional Chinese herbs and is widely cultivated in Southwest China [1]. As a medicine-food homology species, Rhizoma Polygonati Kingiani, the rhizome of *P. kingianum* is commonly used in Chinese medicine and cooking. Clinical cases found that Rhizoma Polygonati Kingiani plays important roles in invigorating Qi, nourishing Yin and moistening lungs as well as tonifying the spleen and kidneys, which are considered to replenish energy, stimulate the secretion of saliva and gastric juices, protect the respiratory system, increase appetite and strengthen immunity [2,3]. Modern pharmacology confirms that these functions are closely related to the content of active substances distributed in the rhizome, in which polysaccharides and steroidal saponins are usually thought to be the most important quality markers [3,4]. 

Saponins are characterized by a skeleton derived of oxidosqualene, consisting of a sugar moiety linked to a triterpenoid (30 carbon atoms) or a steroidal aglycone (27 carbon atoms) [3,5,6]. Steroidal saponins mainly exist in the form of cholestanol, furostanol and spirostanol saponins and are exclusively found in the class of monocotyledonous angiosperms, while triterpenoid saponins are primarily distributed in the class of Magnoliopsida [7,8]. In recent years, steroidal saponins have attracted a great deal of interest due to their diverse pharmacological activities, including anti-inflammatory, vasoprotective, hypocholesterolemic, hypoglycemic, molluscicidal, antifungal and antiparasitic activities [8,9,10]. Steroidal saponins are principally found among monocotyledonous species in the Agavaceeae, Asparagaceae, Dioscoreaceae, Liliaceae and Melanthiaceae families [10,11,12]. The concentrations of steroidal saponins in the rhizomes of *P. kingianum* are in the range of 1~5 mg/g presented in our previous study, and are higher than those of some medicinal plants, e.g., 0.582 mg/g in tuber cortex, 0.228 mg/g in tuber flesh and 29.39 μg/g in the rhizophor of *Dioscorea pseudojaponica* Yamamoto [13].

Unlike manufactured pharmaceuticals, active substances isolated from herbs are chemically complex with multiple components, which are often converted to even more compounds during metabolism after being absorbed into circulation [14]. It is very meaningful to choose appropriate extraction and purification methods before use in preclinical treatment [15], which is a feasible way to uncover the mechanism of active substances. Heating reflux extraction (HRE), Soxhlet extraction and distillation are conventional sample pretreatment techniques, and are widely used for the extraction of active substances, in particular in HRE. However, in fact, there are many drawbacks for these methods, e.g., being time consuming, requiring high purity solvents and presenting low extraction selectivity and efficiency [16,17,18]. Therefore, new methods and assisted strategies need to be created to improve the extraction efficiency and purity. Ultrasound-assisted extraction (UAE) is an efficient method for the extraction of herbal active substances. Evidence showed that compared to Soxhlet extraction, HRE and distillation, the extraction rates of flavonoid, saponin, acetophenone and salvianolic acid B could be significantly improved by UAE in *Nymphaea lotus*, *Eurycoma longifolia*, *Cynanchum bungei* and *Salvia miltiorrhiza* [19,20,21,22]. Ultrasonic treatment generates cavitation, which leads to plant cell wall rupture and thereby facilitates the release of bioactive compounds from plants [23]. The application of the response surface methodology (RSM) is more reasonable and makes it easier to generate parameters of the optimum extraction process.

In this work, we first compared the extractive effect of UAE and HRE for *P. kingianum* steroidal saponins. Then, single-factor experiments and RSM were employed to generate the optimized extractive conditions based on the proper extraction method. Subsequently, the contents of dioscin and diosgenin of *P. kingianum* planted in different areas were determined by high-performance liquid chromatography (HPLC) after extracting total steroidal saponins, of which the contents were used to evaluate the medicinal quality of Rhizoma Polygonati Kingiani. In general, our works can provide support for the study and application of steroidal saponins of *P. kingianum*.

## 2. Results and Discussion

### 2.1. Effect of Different Extraction Methods on the Yield

**Figure 1:**
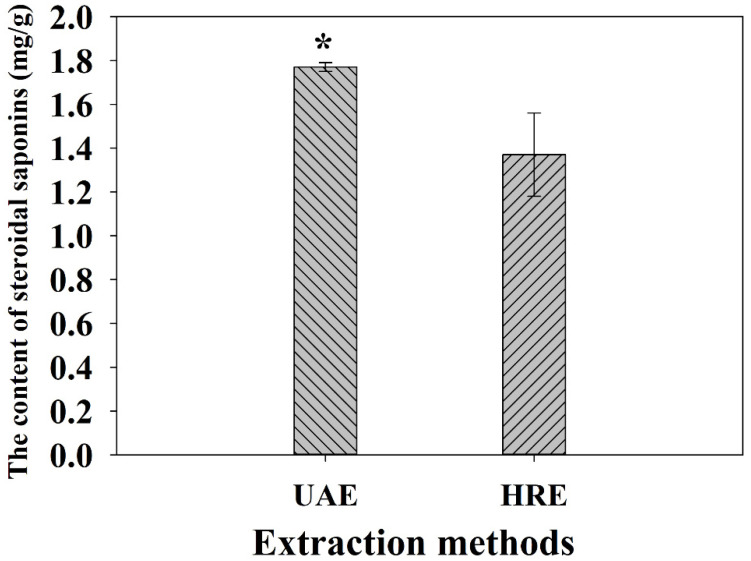
Effect of the extraction methods on the yield of total steroidal saponins. * represents the difference between UAE and HRE, *p* ≤ 0.05. Error bars indicate mean values ± SD, (*n* = 3).

The extraction method is a considerable factor that can significantly influence the extraction efficiency of steroidal saponins in the extraction process. Both UAE and HRE are usually used to extract active substances from plants, any one of which may be chosen according to actual needs. Compared with HRE, UAE has potential advantages, including shorter extraction time, higher extraction efficiency, larger extraction yield and better products with lower cost. Therefore, it is widely used in the extraction of bioactive compounds from natural plants [24,25]. Our results showed that the extraction efficiency of UAE was higher than that of HRE and presented a significant difference (Figure 1). The contents of total steroidal saponins were 1.77 mg/g and 1.47 mg/g, respectively. A possible explanation is that ultrasonic energy increases the internal pressure of the cells in the plant samples, and the increasing power would accelerate cell rupture and promote the destruction of the sample surface [26,27], resulting in the target compounds easily dissolving into the extraction solvent. Therefore, we chose the UAE as the extraction method for the follow-up experiments.

### 2.2. Single-Factor Experiments

#### 2.2.1. Effect of the Liquid-Solid Ratio on the Extraction Yield

**Figure 2:**
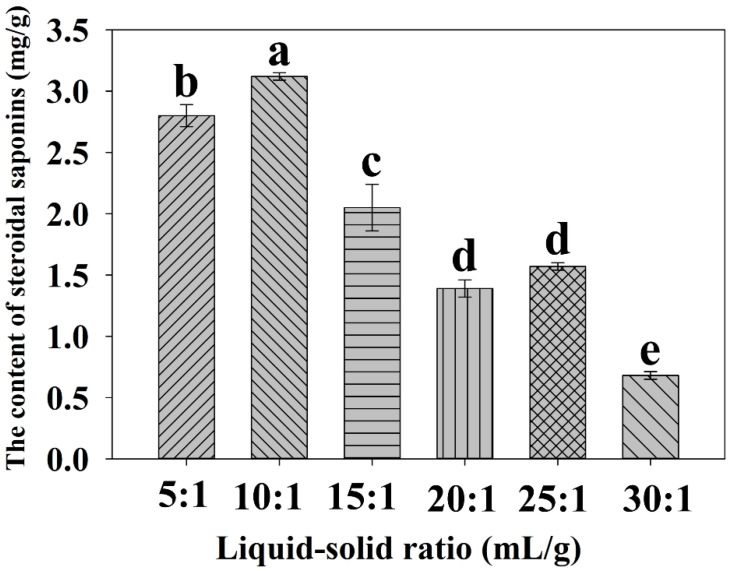
Effect of liquid-solid ratio on the yield of total steroidal saponins. Different lowercase letters represent the difference among treatments of liquid-solid ratios, *p* ≤ 0.05. Error bars indicate mean values ± SD, (*n* = 3).

The liquid-solid ratio significantly affects the solubility of steroidal saponins in aqueous ethanol under the UAE process. Generally, the volume of extraction solvent mainly depends on the amount of the sample [15]. A suitable volume of ethanol solution facilitates the complete dissolution of steroidal saponins from plants [28]. As shown in Figure 2, the yield of crude steroidal saponins increased with increasing extraction solvent from 5- to 10-fold, demonstrating that if the extraction solvent volume is not enough, it is not conducive to the dissolution of saponins from the sample. When the extraction solvent volume increases, the contact area also increases, which would lead to an increase in the extraction efficiency. When the liquid-solid ratio was 10:1 (mL/g), the maximum extraction yield of steroidal saponins was attained with a value of 3.124 mg/g. However, the use of excessive extraction solvent cannot magically improve yield. In vitro, as the liquid-solid ratio was over 10:1 (15:1, 20:1, 25:1, 30:1, mL/g), the yield of total steroidal saponins rose no more and even obviously decreased. This phenomenon could be attributed to the fact that more materials, such as polysaccharides and proteins, were dissolved, hindering the dissolution of saponins [15]. Based on the above results, the liquid-solid ratio was set at 10:1 (mL/g) for other single-factor experiments.

#### 2.2.2. Effect of the Ethanol Concentration on the Extraction Yield

**Figure 3:**
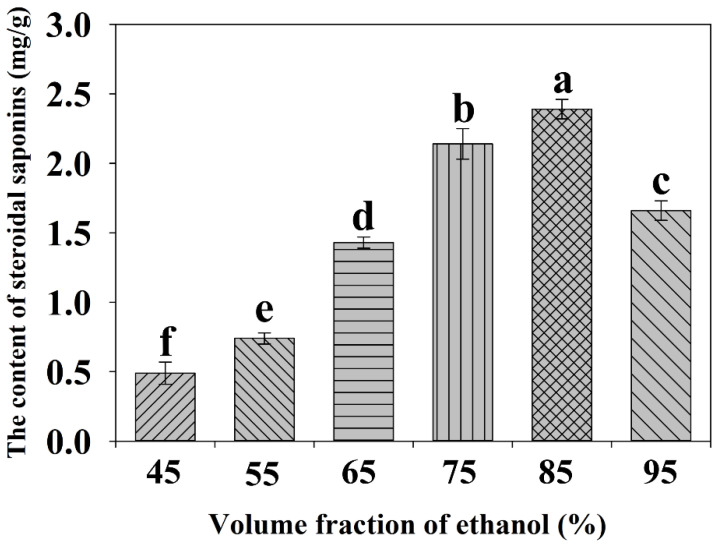
Effect of volume fraction of ethanol on the yield of total steroidal saponins. Different lowercase letters represent the difference among treatments of volume fraction of ethanol, *p* ≤ 0.05. Error bars indicate mean values ± SD, (*n* = 3).

It has been observed that the addition of small amounts of water to the extraction solvent often helps to increase the extraction yield of target compounds from the samples [19,29]. To obtain higher extraction efficiency, the fraction of ethanol was investigated within the range of 45–95% (*v*/*v*) to find the appropriate concentration under the same experimental conditions. As Figure 3 revealed, the yield gradually increased with increasing ethanol fraction from 45% to 85% (*v*/*v*), and the highest extraction yield of steroidal saponins was obtained at 85% aqueous ethanol with a value of 2.39 mg/g. However, further increasing the concentration of ethanol from 85% to 95% resulted in a decrease in the content of steroidal saponins. The reason for this phenomenon might be attributed to the theory of similarity and intermiscibility; if the polarities of the solvent and solute are similar, the solute is easily dissolved from plant cells [30]. As a result, an ethanol fraction of 85% (*v*/*v*) was selected as the optimal volume fraction for the extraction of steroidal saponins.

#### 2.2.3. Effect of the Extraction Time on the Extraction Yield 

**Figure 4:**
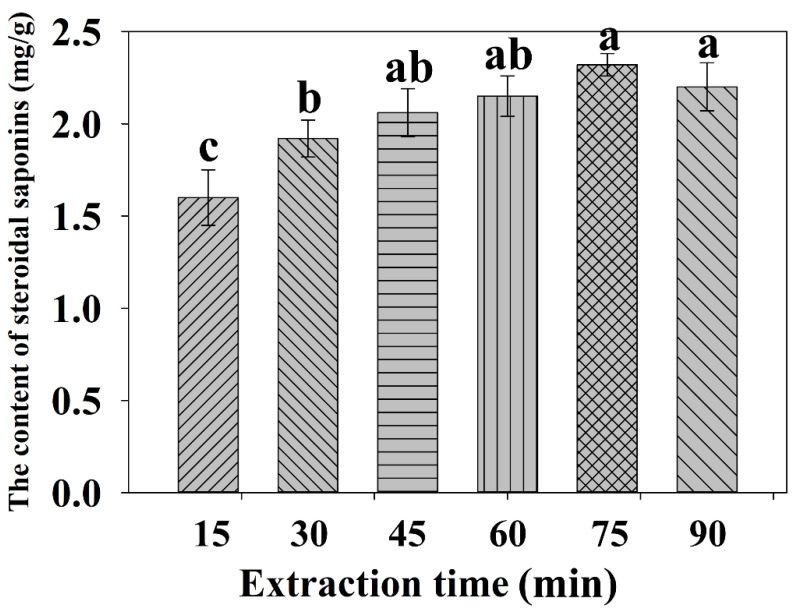
Effect of extraction time on the yield of total steroidal saponins. Different lowercase letters represent the difference among treatments of extraction time, *p* ≤ 0.05. Error bars indicate mean values ± SD, (*n* = 3).

The extraction time is one of the most important factors that affect the extraction yield of steroidal saponins in the UAE process. In theory, the longer the extraction time is, the higher the yield of total steroidal saponins is [31]. As shown in Figure 4, the content of steroidal saponins gradually increased with increasing extraction time from 15 to 75 min. The maximum yield appeared at 75 min with a value of 2.32 mg/g. However, when the extraction time was beyond 75 min, the yield did not improve and even decreased. The reason for this phenomenon might be that the extraction time was too short to completely dissolve total steroidal saponins [32]. An extra amount of time is needed to achieve extraction equilibrium. Further increasing the extraction time may cause negative reactions, triggering the yield decline and wasting too much energy. Therefore, an extraction time of 75 min was appropriate.

#### 2.2.4. Effect of the Extraction Temperature on the Extraction Yield

**Figure 5:**
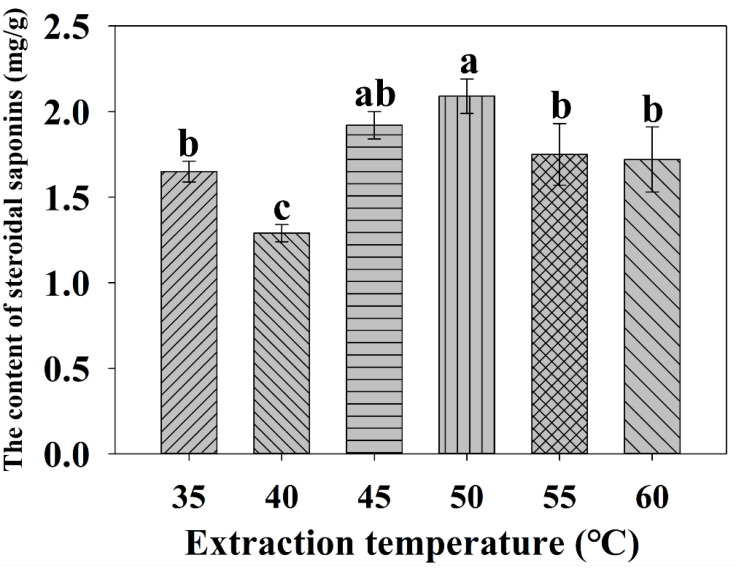
Effect of extraction temperature on the yield of total steroidal saponins. Different lowercase letters represent the difference among treatments of extraction temperature, *p* ≤ 0.05. Error bars indicate mean values ± SD, (*n* = 3).

Extraction temperature is also a crucial variable that impacts the solubility and mass transfer rate of the target compounds in the UAE process [29]. As illustrated in Figure 5, the yield of steroidal saponins increased with the increasing of extraction temperature from 35 to 50 °C, except at 40 °C. The maximum yield reached 2.09 mg/g at 50 °C. As the extraction temperature was above 55 °C, the yield of steroidal saponins presented a slight decrease. This phenomenon can be explained by the fact that a higher temperature would provide a higher kinetic energy to loosen the sample cell structure, greatly intensifying mass transfer phenomena, which leads to a greater rate of diffusion [33,34]. Moreover, high temperatures can increase molecular movement and help to dissolve saponins from plant cells. Taken together, 50 °C was found to be the extraction temperature that produced the highest yield.

#### 2.2.5. Effect of the Numbers of Extractions on the Extraction Yield

**Figure 6:**
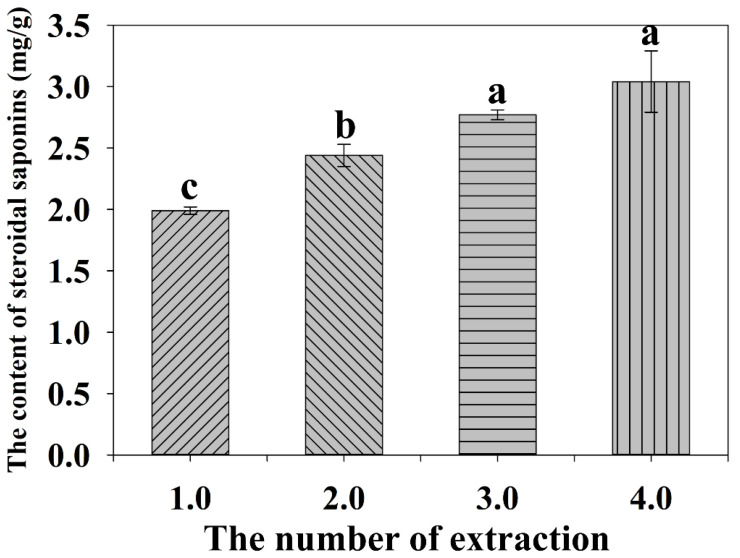
Effect of the number of extractions on the yield of total steroidal saponins. Different lowercase letters represent the difference among treatments of the number of extractions, *p* ≤ 0.05. Error bars indicate mean values ± SD, (*n* = 3).

The effect of the number of extractions on the yields of total steroid saponins was evaluated under the same experimental conditions. As shown in Figure 6, the content of steroid saponins increased as the number of extractions increased from 1 to 4. However, when the number of extractions was more than three extractions, the content of total steroid saponins did not obviously increase any more. Thus, taking the consumption of solvent and extraction efficiency into account, three times were reasonable for extraction in this experiment. 

### 2.3. Optimization of UAE through BBD and RSM

#### 2.3.1. Fitting the Model and Checking Model Adequacy

The results of 29 experimental runs are shown in Table 1 using the Box–Behnken design along with the measured and predicted values for response (Y) of each trial in the experimental design. The second-order model equations for steroidal saponins provided in coded form were expressed as follows:

Y = +4.30 + 0.29A − 0.065B + 0.12C + 0.043D − 0.025AB − 0.13AC − 0.081AD − 0.086BC + 0.075BD + 0.18CD − 1.17A^2^ − 1.42B^2^ − 1.07C^2^ − 0.95D^2^

The roles of the equation were to construct the response surfaces and study the relationship of investigative variables and the responses of steroidal saponins. The magnitude of each coefficient in this equation directly reflected the degree of influence of each factor on the index value, the sign of each represented the direction of influence. Furthermore, ANOVA was employed to determine the adequate and significant matrix, while the R^2^ value was used to judge the adequacy of the models. The ANOVA results are presented in Table 2. The statistical correlation coefficient (R^2^) was greater than 0.9, which indicated the adequacy of the prediction of the experimental results. The *p*-value of this model was < 0.0001, suggesting that the linear and quadratic terms were remarkably significant. Since *p*-values for the lack of fit were insignificant (*p* > 0.05), the validity of the obtained models was confirmed. In addition, the value of Adj R-Squared (Radj^2^) (0.8250) was close to R^2^ (0.9125), implying reasonable adjustment of the model to the experimental data. Therefore, it was concluded that this model could be used to navigate the design space.

#### 2.3.2. Response Surface Analysis

**Figure 7:**
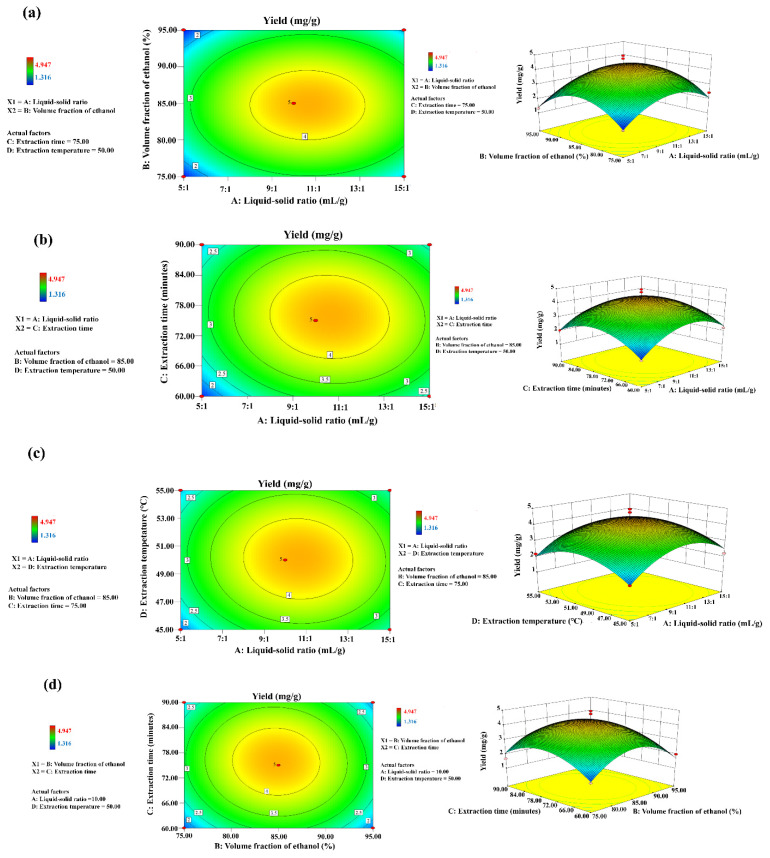

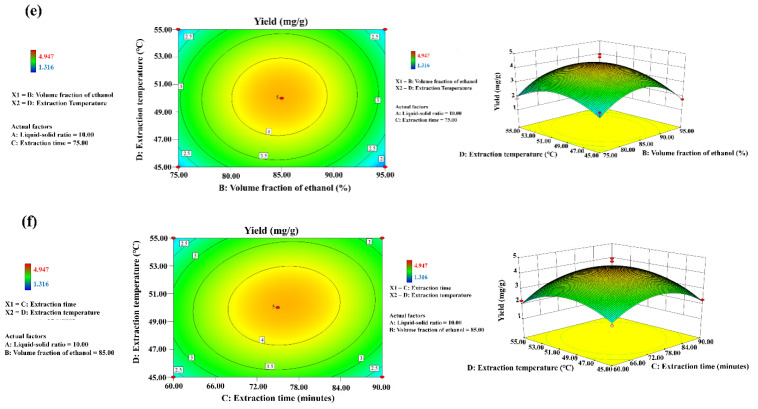
Two-dimensional (2D) contour plots and 3D response surface curves of extraction yield for the interaction effects between liquid-solid ratio and volume fraction of ethanol (**a**), liquid-solid ratio and extraction time (**b**), liquid-solid ratio and extraction temperature (**c**), volume fraction of ethanol and extraction time (**d**), volume fraction of ethanol and extraction temperature (**e**) and extraction time and extraction temperature (**f**).

The three-dimensional (3D) response surface plots provided a method to visualize the relationship between responses and experimental levels of each variable, as well as the type of interactions between two test variables, and to determine the optimum condition of each factor for maximum steroidal saponin yield [35,36]. Figure 7a–f illustrate the 2D contour plots and 3D response surface curves of extraction yield for each pair of parameters when the other independent variable remains constant. The elliptical nature of the 2D contour plot shows mutual interaction between two factors, while circular plots depict negligible mutual interactions [37]. The effect of the liquid-solid ratio and volume fraction of ethanol on the yield of steroidal saponins is shown in Figure 7a. The yield of steroidal saponins increased with the increase in the liquid-solid ratio from 5:1 to 10:1 (mL/g), and the volume fraction of ethanol increased from 45 to 85%. The steroidal saponin yield reached a maximum value when the volume fraction of ethanol was up to 85%, without significant further improvement thereafter. Figure 7b depicts the effect of extraction time and liquid-solid ratio on the yield of steroidal saponins. The maximum yield was obtained at an extraction time of 75 min and decreased on either side of this value. Overly long duration of ultrasound may cause the loss of saponins [38]. In addition, the effects of the interactions between liquid-solid ratio and ultrasound extraction temperature (Figure 7c), volume fraction of ethanol and extraction time (Figure 7d), volume fraction of ethanol and ultrasound extraction temperature (Figure 7e) and extraction time and ultrasound extraction temperature (Figure 7f) on the extraction yield of total steroidal saponins were also analyzed one by one. In conclusion, the optimum extraction technology was generated following a liquid-solid ratio of 10:1 (mL/g), an ethanol concentration of 85% (*v*/*v*), an extraction time of 75 min and an extraction temperature of 50 °C. In addition, the optimal predicted parameters generated by the RSM model were as follows: a liquid-solid ratio (A) of 10.6:1 (mL/g), an ethanol volume fraction of 84.75%, an extraction time (C) of 75.85 min and an ultrasound extraction temperature (D) of 50.10 °C, which were very close to the experimental values with the error values lower than 4.5%, indicating that the designed model was very accurate and reliable.

### 2.4. Method Validation with HPLC

#### 2.4.1. Calibration Curves and Linear Range

The calibration curves and linear ranges of dioscin and diosgenin were generated and are listed in Table 3, of which the equations of linear regressions were y = 18.895x − 6.272 (R^2^ = 0.9999; linearity range, 14.4 × 10^−3^~86.4 × 10^−3^ mg) for dioscin and y = 41.514x + 15.031 (R^2^ = 0.9958; linearity range, 1.95 × 10^−3^~11.7 × 10^−3^ mg) for diosgenin, respectively.

#### 2.4.2. Precision

The precision results of HPLC for determining dioscin and diosgenin are shown in Table 4. The precision of dioscin and diosgenin to be tested was 1.79% and 4.27%, respectively, demonstrating that the precision was good for this method.

#### 2.4.3. Extraction Recovery Rate

The extraction recovery rate of this method is shown in Table 5. The results showed that the recovery rates of the assay method for dioscin and diosgenin were 122.2% and 110.6%, respectively, meeting the requirements of guiding principles for biological sample analysis of the recovery. Thus, the extraction recovery of this method was reliable.

#### 2.4.4. Test of Biological Sample Stability

To test the stability of the biological sample, we repeated the experiment 6 times under the same conditions. The results showed that the RSDs of dioscin and diosgenin were 1.77% and 3.80%, respectively, as shown in Table 6. It was believed that the biological sample in this experiment was stable in this experiment.

#### 2.4.5. Contents of Dioscin and Diosgenin in the Rhizomes of *P. kingianum* Planted in Different Areas

**Figure 8:**
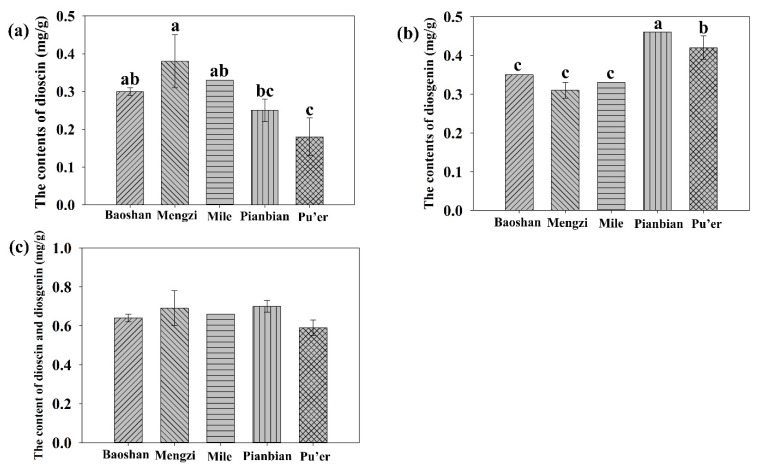
The contents of dioscin (**a**), diosgenin (**b**) and the sum (dioscin and diosgenin) (**c**) in the rhizomes of *Polygonatum kingianum* planted in five different areas, including Baoshan Prefecture (BS), Mengzi Prefecture (MZ), Mile Prefecture (ML), Pingbian Prefecture (PB) and Pu’er Prefecture (PE). Different lowercase letters represent the difference of contents of dioscin, diosgenin or the sum among the five samples, *p* ≤ 0.05. There is no difference among bars of (**c**). Error bars indicate mean values ± SD, (*n* = 3). BS: Baoshan Prefecture; MZ: Mengzi Prefecture; ML: Mile Prefecture; PB: Pingbian Prefecture; PE: Pu’er Prefecture.

On the basis of the optimal extraction process, the contents of dioscin and diosgenin in the rhizomes of *P. kingianum* from five different sampling plots were analyzed using HPLC with the method described above. As shown in Figure 8a, the dioscin content of MZ was the highest with a value of 0.38 mg/g, followed by ML (0.33 mg/g), BS (0.30 mg/g), PB (0.25 mg/g) and PE (0.18 mg/g). A significant difference was observed between MZ/ML/BS and PE. However, there were no obvious differences among the MZ, ML and BS. For diosgenin content in Figure 8b, the value of PB (0.46 mg/g) was the highest, followed by the value of 0.42 mg/g of PE with a nonsignificant difference. The diosgenin contents of BS, MZ and ML were very close and were significantly less than those of PB or PE. The sum of dioscin and diosgenin showed that the content of PB was the highest, but there was no significant difference among the five samples (Figure 8c).

## 3. Materials and Methods

### 3.1. Reagents and Apparatus 

Vanillic aldehyde (CAS: 121-33-5, AR), ethanol (CAS: 64-17-5, AR) and sodium hydroxide (CAS: 1310-73-2, AR) were purchased from Tianjin Guangfu, Fine Chemical Research Institute (Tianjin, China). Glacial acetic acid, *n*-butanol (CAS: 71-36-3, AR) and perchloric acid (CAS: 7601-90-3, AR) were purchased from Guangdong Guanghua Technology Co., Ltd. (Guangdong, China). All saponin standard substances (purity ≥ 99%) (CAS: 512-04-9, AR; CAS: 19057-60-4, AR) were obtained from Beijing Solebo Technology Co., Ltd. (Beijing, China). Instrument equipment mainly used in the experiment included the following: An ultraviolet-visible spectrophotometer (Shimadzu UV-2550, Shanghai, China), a swing-type high-speed crusher (Linda DFY-800D, Wenling, China), an ultrasonic cleaner (SK72010HP, China), a rotary evaporator (Aika HB10 SO96, Guangzhou, China), an HPLC system (Agilent 1260, Palo Alto, CA, USA) and an electric constant temperature blast-drying oven (Yuejin DHG-9420A, Shanghai, China). 

### 3.2. Plant Material

Plant materials used in the experiments were purchased from rural markets of different Prefectures in Yunnan Province, China, which were authenticated as *P. kingianum* by Prof. Rong-Hua Zhao (Yunnan University of Chinese Medicine). These plants were cultivated for 3 years. The raw material for the extraction process was a dried rhizome of *P. kingianum* planted in Mengzi Prefecture. Samples used for quality assessment were sampled from Mile Prefecture (ML), Pingbian Prefecture (PB), Mengzi Prefecture (MZ), Baoshan Prefecture (BS) and Pu’er Prefecture (PE), which were primary planting areas.

### 3.3. Comparison of the Extraction Methods

The extraction effects of UAE and HRE were compared by determining the contents of total steroidal saponins; both are often used to extract saponin. UAE was performed on an ultrasonic cleaner (SK72010HP, Shanghai, China), while HRE was conducted by a glass reflux unit. For UAE, 10.000 g dried sample powder was precisely weighed and placed into a distilling flask containing 100 mL of 85% (*v*/*v*) ethanol solution (ethanol:water 85:15) and extracted under a 53 kHz frequency and 50 °C water bath for 1 h [39,40]. This was extracted twice with residue. The process of HRE was water bath reflux with 85% ethanol for 3 h at 80 °C and the process was repeated two more times with residue [41]. The extracts were filtered and concentrated via vacuum rotary evaporation. Then, the extractum was extracted with 20 mL *n*-butanol, and it was repeated again with residue. The *n*-butanol solution containing steroidal saponins was centrifuged for 10 min at 3500 r/min. A crude extractum was obtained after removing the organic solvent, and was transferred into a 5 mL volumetric flask by dissolving in ethanol, filling it to constant volume with ethanol.

### 3.4. Determination of Total Steroidal Saponins

Studies have shown that two main types of steroidal saponins in the rhizomes of *P. kingianum*, furostanol and spirostanol saponin, can be hydrolyzed to diosgenin by dilute acid [42,43,44]. The total content of steroidal saponins was determined in the form of diosgenin using ultraviolet spectrophotometry as described by Liu and Bi [43,44]. Vanillin-glacial acetic acid and perchloric acid (1:4) were taken as color development reagents, and a maximum absorption peak emerged at 545 nm by reaction with diosgenin hydrolyzed from steroidal saponins. Firstly, a volume of 0.4 mL prepared crude extract or ethanol (CK) was transferred into a colorimetric tube, accelerating volatilization of the solvent via boiling water bath. Then, 0.20 mL of freshly prepared 10% vanillin-glacial acetic acid solution and 0.80 mL perchloric acid reagent were added, mixed and incubated at 60 °C for 10 min. Then, 5.0 mL glacial acetic acid was added after being kept in an ice-water bath for 5 min. Subsequently, the absorbance of the resulting solution was determined at 545 nm. A standard curve of y = 11.794x + 0.0444 (R^2^ = 0.9943) with a linearity range of 0.5–8.5 mg/g was calculated, where x was the concentration of diosgenin (mg/g) and y was the absorbance value.

### 3.5. Single-Factor Experiment

In total, 5 factors were chosen in order to investigate the roles that affected the extraction yield of steroidal saponins, including the liquid-solid ratio, ethanol concentration, extraction time, extraction temperature and number of extractions. For the independent variables in the experimental design, the conditions of extraction methods were as follows: liquid-solid ratio (mL/g): 5:1, 10:1, 15:1, 20:1, 25:1 and 30:1; volume fraction of ethanol (%): 45, 55, 65, 75, 85 and 95; extraction time (minutes): 15, 30, 45, 60, 75 and 90; extraction temperature (°C): 35, 40, 45, 50, 55 and 60; number of extractions: 1, 2, 3 and 4 [45]. The content of total steroidal saponins was determined as described above. All samples were prepared in triplicate for experimental analysis, and each measurement was performed three times.

### 3.6. Response Surface Methodology

As an effective statistical method, RSM is usually used to generate the optimal experimental conditions, e.g., an optimization extraction process. According to the results of single-factor experiments, the liquid-solid ratio (A), ethanol concentration (B), extraction time (C) and ultrasound extraction temperature (D) were selected as independent variables for RSM, while the concentration of steroidal saponins was used as the response (dependent) variable (Y). The optimization was carried out through a three-level, four-factor Box–Behnken design (BBD) project consisting of 29 experimental runs including five replicates at the central point. The three different levels were set as −1 (low), 0 (medium) and +1 (high). The coded and actual values of the experimental factors for the BBD are shown in Table 7. The extraction procedure and determination methods were the same as those described above. Analysis of variance (ANOVA) was carried out to determine individual linear, quadratic and interaction regression coefficients using Design-Expert software version 8.0.6 (Stat-Ease, Inc.). The coefficient of determination (R^2^) was used to assess the fitness of the quadratic polynomial equation to the experimental responses, and the significance of the model and independent variables was evaluated by computing the F value at a *p* value < 0.05.

### 3.7. HPLC Analysis of Dioscin and Diosgenin in P. kingianum

On the basis of the optimal extraction conditions, crude extracts of steroidal saponins were obtained from the rhizome of *P. kingianum* planted in different areas. Subsequently, HPLC was employed to precisely analyze the concentrations of dioscin and diosgenin, of which the dioscin and diosgenin were acid-hydrolyzed from steroidal saponins. The acid-hydrolysis method was used as described by Zhao [46]. The hydrolysis product was concentrated via vacuum rotary evaporation and then dissolved in methanol and filtered through a 0.45 μm membrane filter before HPLC-UV analysis. An Agilent 1260 HPLC system (Agilent Technologies, USA) equipped with a UV detector was used to determine the concentrations of dioscin and diosgenin. The Agilent SB-C_18_ column (5 μm, 4.6 × 150 mm) was maintained at 28 °C. The mobile phase consisted of A (acetonitrile) and B (water) with an elution gradient of 0–40 min, 80% A and 20% B. The flow rate and UV detection wavelength were set at 1 mL/min and 203 nm, respectively, and the injection volume was 15 μL. The chromatographic peaks of dioscin and diosgenin were confirmed by comparing retention times and UV spectra with reference standard (*Chinese Pharmacopoeia, 2020*). 

A seven-point standard curve (0–100 µg) was constructed using standard substances of dioscin and diosgenin, of which the equations of linear regression were y = 18.895x − 6.272 (R^2^ = 0.9999; linearity range, 14.4 × 10^−3^~86.4 × 10^−3^ mg) for dioscin and y = 41.514x + 15.031 (R^2^ = 0.9958; linearity range, 1.95 × 10−3~11.7 × 10^−3^ mg) for diosgenin. In addition, method validation was carried out as described by Wang, including precision, recovery rates and stability test [15]. 

### 3.8. Statistical Analysis

Data were processed with Microsoft Excel software and SPSS 17.0, in which Duncan’s multiple range test was used for ANOVA. All data were performed in triplicate and expressed with the mean ± standard deviation (SD) (*n* = 3). In addition, figures were drawn with GraphPad Prism 5, while Design-Expert 8.0.6 software (Stat-Ease, Inc., Chicago, IL, USA) was applied for BBD.

## 4. Conclusions

In this study, UAE was proven to be an efficient extraction method for *P. kingianum* steroidal saponins, and the extraction yield was significantly affected by the liquid-solid ratio. The optimal UAE technology was generated via single-factor experiments and RSM following a liquid-solid ratio of 10:1 (mL/g), an ethanol concentration of 85% (*v*/*v*), an extraction time of 75 min, an extraction temperature of 50 °C and three extractions, of which these parameters were in line with the predicted values. Considering only dioscin and diosgenin, the quality of *P. kingianum* planted at five sample plots presented non-significant difference. However, the content of diosgenin in Pingbian Prefecture (PB) was higher than that of the other four areas with a value of 0.46 mg/g. Taken together, the optimal UAE technology for *P. kingianum* steroidal saponins was determined via RSM. The quality evaluation revealed that there was a non-significant difference among *P. kingianum* planted in different areas based on the contents of the sum of dioscin and diosgenin. This work has important reference value for the exploitation and utilization of *P. kingianum.*

## Figures and Tables

**Table 1:** Box–Behnken design matrix with coded variables and measured and predicted values.

Run	Factors				Response	
	Liquid- Solid Ratio	Volume Fraction of Ethanol (%)	Extraction Time (min)	Ultrasound Extraction Temperature (°C)	Total Steroid Saponins (mg/g)	
					Measured	Predicted
1	5	75	75	50	1.43	1.46
2	15	75	75	50	2.44	2.09
3	5	95	75	50	1.32	1.38
4	15	95	75	50	2.23	1.91
5	10	85	60	40	2.14	2.30
6	10	85	90	45	2.28	2.18
7	10	85	60	55	2.20	2.02
8	10	85	90	55	3.08	2.63
9	5	85	75	45	1.77	1.76
10	15	85	75	45	2.20	2.51
11	5	85	75	55	2.17	2.01
12	15	85	75	55	2.28	2.43
13	10	75	60	50	1.59	1.67
14	10	95	60	50	1.99	1.71
15	10	75	90	50	1.67	2.09
16	10	95	90	50	1.72	1.78
17	5	85	60	50	1.48	1.52
18	15	85	60	50	2.22	2.36
19	5	85	90	50	2.03	2.03
20	15	85	90	50	2.28	2.35
21	10	75	75	45	2.37	2.02
22	10	95	75	45	1.76	1.74
23	10	75	75	55	1.80	1.96
24	10	95	75	55	1.49	1.98
25	10	85	75	50	3.86	4.30
26	10	85	75	50	4.01	4.30
27	10	85	75	50	4.95	4.30
28	10	85	75	50	3.94	4.30
29	10	85	75	50	4.75	4.30

**Table 2:** ANOVA of response surface model and predicted results for response of four analytes.

Source	Total Steroid Saponins		
	Coefficient	F-Value	*p*-Value
Model	4.30	10.43	<0.0001 **
A	0.29	6.02	0.0278
B	−0.065	0.31	0.5892
C	0.12	1.06	0.3199
D	0.043	0.13	0.7246
AB	−0.025	0.014	0.9065
AC	−0.13	0.39	0.5412
AD	−0.081	0.15	0.7001
BC	−0.086	0.18	0.6791
BD	0.075	0.13	0.7196
CD	0.18	0.81	0.3840
A^2^	−1.17	52.97	<0.0001 **
B^2^	−1.42	78.15	<0.0001 **
C^2^	−1.07	43.94	<0.0001 **
D^2^	−0.95	35.12	<0.0001 **
Lack of fit		0.51	0.8235
C.V. %	17.11		
R^2^	0.9125		
Pred.R^2^	0.65676		
Adj-R^2^	0.8250		

** represent the linear and quadratic terms were remarkably significant.

**Table 3:** Calibration curves of dioscin and diosgenin using HPLC (*n* = 6).

Analytes	Calibration Curves	Linear Range (mg)	R^2^
Dioscin	y = 18.895x − 6.272	14.4 × 10^−3^–86.4 × 10^−3^	0.9999
Diosgenin	y = 41.514x + 15.031	1.95 × 10^−3^–11.7 × 10^−3^	0.9958

**Table 4:** Results of experimental precision (*n* = 6).

Analytes	Peak Area						Average	SD	RSD (%)
Dioscin	1163.7	1152.5	1117.6	1111.3	1139.2	1126.9	1135.2	20.38	1.79
Diosgenin	225.8	207.3	228.2	220.9	211.7	230.8	220.8	9.43	4.27

**Table 5:** Recovery rates of dioscin and diosgenin using HPLC (*n* = 6).

NO.	Sampling Quantity (g)		Sample Content (μg)		Added Quantity (μg)		Measured Quantity (μg)		Rate of Recovery (%)		X		RSD (%)	
	A	B	A	B	A	B	A	B	A	B	A	B	A	B
1	20.0004	20.0004	1939.71	514.88	1575	444	3879.43	1029.76	123.16	115.96	122.2	110.6	2.9	4.7
2	20.0010	20.0010	1960.88	501.49	1575	444	3921.77	1002.98	124.50	112.95				
3	20.0031	20.0031	1960.04	467.48	1575	444	3920.07	934.95	124.45	105.29				
4	20.0002	20.0002	1932.09	462.56	1575	444	3864.18	925.12	122.67	104.18				
5	20.0015	20.0015	1945.01	515.84	1575	444	3890.01	1031.69	123.49	116.18				
6	20.0031	20.0031	1814.81	485.30	1575	444	3629.63	970.60	115.23	109.30				

Note, A and B represent dioscin and diosgenin, respectively.

**Table 6:** Results of stability test (*n* = 6).

Analytes	Peak Area						Average	SD	RSD (%)
Dioscin	425.7	419.2	413.2	412.6	410.6	404.4	414.3	7.35	1.77
Diosgenin	207.9	200.3	223.2	217.0	207.3	213.4	211.5	8.08	3.8

**Table 7:** Variables and their levels used in the experiments.

Independent Variables	Symbol	Levels		
		−1	0	1
Liquid-solid ratio (mL/g)	A	5:1	10:1	15:1
Volume fraction of ethanol (%)	B	75	85	95
Extraction time (minutes)	C	60	75	90
Ultrasound extraction temperature (°C)	D	45	50	55

## Data Availability

Not applicable.

## References

[1] Liu J.J., Si J.P. (2018). Herbal textual research on Chinese medicine “Huangjing” (*Polygonati Rhizoma*) and some enlightenments. China J. Chin. Mater. Med..

[2] Singh B., Singh J.P., Singh N., Kaur A. (2017). Saponins in pulses and their health promoting activities: A review. Food. Chem..

[3] Zhao P., Cheng C.C., Li X., Gao Q.Z., Huang L.Q., Xiao P.G., Gao W.Y. (2017). The genus *Polygonatum*: A review of ethnopharmacology, phytochemistry and pharmacology. J. Ethnopharmacol..

[4] Yan H.L., Lu J.M., Wang Y.F., Gu W., Yang X.X., Yu J. (2017). Intake of total saponins and polysaccharides from *Polygonatum kingianum* affects the gut microbiota in diabetic rats. Phytomedicine.

[5] KEGG (2017). Kyoto Encyclopedia of Genes and Genomes. http://www.genome.jp/kegg/.

[6] Vincken J.P., Heng L., de Groot A., Gruppen H. (2007). Saponins, classification and occurrence in the plant kingdom. Phytochemistry.

[7] Faizal A., Geelen D. (2013). Saponins and their role in biological processes in plants. Phytochem. Rev..

[8] Sparg S.G., Light M.E., Van S.J. (2004). Biological activities and distribution of plant saponins. J. Ethnopharmacol..

[9] Osbourn A.E., Qi X., Townsend B., Qin B. (2003). Dissecting plant secondary metabolism constitutive chemical defences in cereals. New. Phytol..

[10] Podolak I., Galanty A., Sobolewska D. (2010). Saponins as cytotoxic agents: A review. Phytochem. Rev..

[11] Sahu N.P., Banerjee S., Mondal N.B., Mandal D. (2008). Steroidal saponins. Fortschritte der Chemie Organischer Naturstoffe. Progress in the Chemistry of Organic Natural Products.

[12] Yang Z.Y., Yang L.F., Liu C.K., Qin X.J., Liu H.Y., Chen J.H., Ji Y.H. (2019). Transcriptome analyses of *Paris polyphylla* var. chinensis, *Ypsilandra thibetica*, and *Polygonatum kingianum* characterize their steroidal saponin biosynthesis pathway. Fitoterapia.

[13] Lin J.T., Yang D.J. (2008). Determination of steroidal saponins in different organs of yam (*Dioscorea pseudojaponica Yamamoto*). Food. Chem..

[14] Qiao X., Ye M., Xiang C., Wang Q., Liu C.F., Miao W.J., Guo D.A. (2012). Analytical strategy to reveal the in vivo process of multi-component herbal medicine: A pharmacokinetic study of licorice using liquid chromatography coupled with triple quadrupole mass spectrometry. J. Chromatogr. A..

[15] Wang Q.Y., Dong X., Yang J., Hu Y.H., Peng L.Q., Zheng H., Cao J. (2020). Vesicle based ultrasonic-assisted extraction of saponins in *Panax notoginseng*. Food. Chem..

[16] Yang Y.C., Wei M.C., Huang T.C., Lee S.Z., Lin S.S. (2013). Comparison of modified ultrasound-assisted and traditional extraction methods for the extraction of baicalin and baicalein from *Radix Scutellariae*. Ind. Crop. Prod..

[17] Yang B.Y., Zhang M.Y., Weng H.Y., Xu Y., Zeng L.H. (2020). Optimization of ultrasound assisted extraction (UAE) of kinsenoside compound from *Anoectochilus roxburghii* (Wall.) Lindl by response surface methodology (RSM). Molecules.

[18] Nguyen V.T., Pham H.N.T., Bowyer M.C., Altena I.A., Scarlett C.J. (2016). Influence of solvents and novel extraction methods on bioactive compounds and antioxidant capacity of *Phyllanthus amarus*. Chem. Pap..

[19] Dong J., Liu Y.B., Liang Z.S., Wang W.L. (2010). Investigation on ultrasound-assisted extraction of salvianolic acid B from *Salvia miltiorrhiza* root. Ultrason. Sonochem..

[20] Elhag H.E.E.A., Naila A., Ajit A., Aziz B.A., Sulaiman A.Z. (2018). Sequential extraction of saponins from *Eurycoma longifolia* roots by water extraction and ultrasound-assisted extraction. Mater. Today Proc..

[21] Sun Y.S., Liu Z.B., Wang J.H., Yan S.F., Li B.Q., Xu N. (2013). Aqueous ionic liquid based ultrasonic assisted extraction of four acetophenones from the Chinese medicinal plant *Cynanchum bungei* Decne. Ultrason. Sonochem..

[22] Tungmunnithum D., Drouet S., Kabra A., Hano C. (2020). Enrichment in antioxidant flavonoids of stamen extracts from *Nymphaea lotus* L. using ultrasonic-assisted extraction and macroporous resin adsorption. Antioxidants.

[23] Chua L.S. (2013). A review on plant-based rutin extraction methods and its pharmacological activities. J. Ethnopharmacol..

[24] Charalampos P., Michael K. (2008). Application of microwave-assisted extraction to the fast extraction of plant phenolic compounds. Food. Sci. Tech..

[25] Kamaljit V., Raymond M., Lloyd S., Darren B. (2008). Application and opportunities for ultrasound assisted extraction in the food industry: A review. Innov. Food. Sci. Emerg..

[26] Pham H.N.T., Vuong Q.V., Bowyer M.C., Scarlett C.J. (2018). Ultrasound-assisted extraction of *Catharanthus roseus* L. G. Don (*Patricia White* cultivar) stem for maximizing saponin yield and antioxidant capacity. J. Food. Process. Preserv..

[27] Zhang H.X., Birch J., Xie C.N., Yang H.Y., Bekhit A.E.D. (2019). Optimization of ultrasound assisted extraction method for phytochemical compounds and in-vitro antioxidant activity of New Zealand and China Asparagus cultivars (*officinalis* L.) roots extracts. Food. Chem..

[28] Herodez S.S., Hadolin M., Skerget M., Knez Z. (2003). Solvent extraction study of antioxidants from Balm (*Melissa officinalis* L.) leaves. Food. Chem..

[29] Ren Y., Chen Y., Hu B.H., Wu H., Lai F., Li X.F. (2015). Microwave-assisted extraction and a new determination method for total steroid saponins from *Dioscorea zingiberensis* CH. Wright. Steroids.

[30] Gribova N.Y., Filippenko T.A., Nikolaevskii A.N., Belaya N.I., Tsybulenko A.A. (2008). Optimization of conditions for the extraction of antioxidants from solid parts of medicinal plants. J. Anal. Chem..

[31] Hu T., Guo Y.Y., Zhou Q.F., Zhong X.K., Zhu L., Piao J.H., Chen J., Jiang J.G. (2012). Optimization of ultrasonic-assisted extraction of total saponins from *Eclipta prostrasta* L. using response surface methodology. J. Food. Sci..

[32] Xu L.J., Fang H.T., Shen J.L., Zhang J., Liu S.S., Ding X.M., Zhang M.H., Tang H.Y., Tang X. Optimization of extraction of flavonoids from *Pteridum aquilinum* var. latiusculum. Proceedings of the International Conference on Remote Sensing, Environment and Transportation Engineering.

[33] Allaf T., Tomao V., Ruiz K., Chemat F. (2013). Instant controlled pressure drop technology and ultrasound assisted extraction for sequential extraction of essential oil and antioxidants. Ultrason. Sonochem..

[34] Belwal T., Huang H., Li L., Duan Z.H., Zhang X.B., Aalim H., Luo Z.S. (2019). Optimization model forultrasonic-assisted and scale-up extraction of anthocyanins from Pyrus communis ‘starkrimson’ fruit peel. Food. Chem..

[35] Chen W., Wang W.P., Zhang H.S., Huang Q. (2012). Optimization of ultrasonic-assisted extraction of water-soluble polysaccharides from *Boletus edulis* mycelia using response surface methodology. Carbohyd. Polym..

[36] Pan G.Y., Yu G.Y., Zhu C.H., Qiao J.L. (2012). Optimization of ultrasound-assisted extraction (UAE) of flavonoids compounds (FC) from hawthorn seed (HS). Ultrason. Sonochem..

[37] Parkhey P., Ram A.K., Diwan B., Eswari J.S., Gupta P. (2020). Artificial neural network and response surface methodology: A comparative analysis for optimizing rice straw pretreatment and saccharification. Prep. Biochem. Biotech..

[38] Li H.Y., Deng Z.Y., Wu T., Liu R.H., Loewen S., Tsao R. (2012). Microwave-assisted extraction of phenolics with maximal antioxidant activities in tomatoes. Food. Chem..

[39] Zhang L., Chang Q., Wang Y., Liu H.B., Song Z.X. (2019). Optimization of extraction process of total polysaccharides, saponins and flavonoids in stem of Huangjing (Polygonatum sibiricum) by response surface method. Guid. J. Tradit. Chin. Med. Pharm..

[40] Huang J.Y., Cui F.Y., Zheng S.J., Jiang J.X., Yang J.Y., Wang Z.G., Yu C.M., Yang B. (2021). Extraction process of total saponins and total flavonoids from Polygonati Rhizoma by response surface optimization and its application in contents comparison from Polygonati rhizoma in different origin. Acta Chin. Med. Pharmacol..

[41] Wang P., Ma C.Y., Chen S.W., Zhu S., Lou Z.S., Wang H.X. (2014). Ionic liquid-based ultrasonic/microwave-assisted extraction of steroidal saponins from *Dioscorea zingiberensis* C. H. Wright. Trop. J. Pharm. Res..

[42] Yang H., Yin H.W., Wang X.W., Li Z.H., Shen Y.P., Jia X.B. (2015). In situ pressurized biphase acid hydrolysis, a promising approach to produce bioactive diosgenin from the tubers of *Dioscorea zingiberensis*. Pharm. Mag..

[43] Bi Y.W., Yang Y.H., Gong J.H., Chen B.F., Liu Z.B. (2010). Determination of polysaccharide and diosgenin in *Polygonatum* and *Polygonatum multiflorum*. J. Chang. Univ. Tradit. Chin. Med..

[44] Liu F.Y. (2017). Establishment of Tissue Culture System of *Polygonatum multiflorum* and Extraction of Steroidal Saponins. Master’s Thesis.

[45] Zhao L.R., Luo H., Xiang Y.L., Sun H.J., Mei G.L., Fang C.W. (2018). Optimization of microwave extracting total saponins from rhizoma Polygonati by Box-Benhken response surface method. Mod. Chin. Med..

[46] Zhao P.P., Li B.M., He L.Y. (1987). Studies on the method of determination of combined sugars in glycosides. Acta Pharm. Sin..

